# Treatment expectancy, working alliance, and outcome of Trauma-Focused Cognitive Behavioral Therapy with children and adolescents

**DOI:** 10.1186/s13034-018-0223-6

**Published:** 2018-03-05

**Authors:** Veronica Kirsch, Ferdinand Keller, Dunja Tutus, Lutz Goldbeck

**Affiliations:** Department of Child and Adolescent Psychiatry and Psychotherapy, University of Ulm, Steinhoevelstr. 5, 89075 Ulm, Germany

**Keywords:** Caregiver, Children and adolescents, Collaboration, Posttraumatic stress symptoms, TF-CBT, Treatment expectancy, Working alliance

## Abstract

**Background:**

It has been shown that positive treatment expectancy (TE) and good working alliance increase psychotherapeutic success in adult patients, either directly or mediated by other common treatment factors like collaboration. However, the effects of TE in psychotherapy with children, adolescents and their caregivers are mostly unknown. Due to characteristics of the disorder such as avoidant behavior, common factors may be especially important in evidence-based treatment of posttraumatic stress symptoms (PTSS), e.g. for the initiation of exposure based techniques.

**Methods:**

TE, collaboration, working alliance and PTSS were assessed in 65 children and adolescents (age *M* = 12.5; *SD* = 2.9) and their caregivers. Patients’ and caregivers’ TE were assessed before initiation of Trauma-Focused Cognitive Behavioral Therapy (TF-CBT). Patients’ and caregivers’ working alliance, as well as patients’ collaboration were assessed at mid-treatment, patients’ PTSS at pre- and post-treatment. Path analysis tested both direct and indirect effects (by collaboration and working alliance) of pre-treatment TE on post-treatment PTSS, and on PTSS difference scores.

**Results:**

Patients’ or caregivers’ TE did not directly predict PTSS after TF-CBT. Post-treatment PTSS was not predicted by patients’ or caregivers’ TE via patients’ collaboration or patients’ or caregivers’ working alliance. Caregivers’ working alliance with therapists significantly contributed to the reduction of PTSS in children and adolescents (post-treatment PTSS: β = − 0.553; *p* < 0.001; PTSS difference score: β = 0.335; *p* = 0.031).

**Conclusions:**

TE seems less important than caregivers’ working alliance in TF-CBT for decreasing PTSS. Future studies should assess TE and working alliance repeatedly during treatment and from different perspectives to understand their effects on outcome. The inclusion of a supportive caregiver and the formation of a good relationship between therapists and caregivers can be regarded as essential for treatment success in children and adolescents with PTSS.

## Background

For decades of psychotherapy research, there has been an ongoing—and often lively—debate to find out if common ingredients of a treatment, like, e.g. expectations of improvement, or more specific elements—like, e.g. exposure in trauma-therapy—are responsible for psychotherapeutic success. This argument has led to numerous studies, with the question of how to deliver the most efficacious treatment still unanswered [1]. Thus, researchers have recently begun to integrate both sides into one comprehensive model, reflecting the need for a more differentiated adaptation of common and specific treatment aspects, psychiatric disorders and the individuality of the patient, to improve therapeutic success [2, 3].

This integrative approach seems helpful in the context of post-traumatic stress disorder (PTSD), a severe and chronic psychiatric condition leading to profound psychosocial impairment. For instance, both specific and common factors were reported to have substantial and unique impact on treatment success in adults with PTSD [4, 5]. Furthermore, the interplay between these factors may depend on the individual trauma history of the patient and his/her posttraumatic stress symptoms (PTSS; [6]). Traumatic experiences—especially interpersonal ones like sexual or physical violence—often lead to a loss of confidence in oneself, others and the world, so that the affected persons may have difficulties in establishing therapeutic relationships. Moreover, the ability to anticipate a positive outcome is decreased; therefore, patients might become less responsive to common factors. For such patients, evidence based treatment techniques, like exposure to trauma related stimuli, may be more important than common factors in order to facilitate symptom reduction [6, 7]. On the other hand, a good relationship with the therapist and positive outcome expectations seem essential prerequisites to engage patients in challenging exposure techniques, especially patients showing avoidant behavior as usual in PTSD [8], highlighting the importance of common factors.

One of the first advocates for acknowledging the importance of common treatment aspects [9] claimed, that positive outcome expectations were one of the most important factors in symptom change. However, research regarding treatment expectancy (TE), i.e. prognostic beliefs about the consequences of engaging in treatment [10] is rare. For adult patients, the clinical relevance of TE is supported by a meta-analysis indicating a small significant positive effect (*d* = 0.24) on treatment outcome regarding different mental disorders [10]. The authors found that better outcome expectations, assessed at an early stage of treatment, were associated with higher symptom change after treatment completion.

Due to developmental factors and the triangulated relationship with caregivers, findings from research with adults cannot be directly applied to children and adolescents. First of all, their capacity for discerning and verbalizing internal states, as well as—in consequence—TE is limited, and differs from grown-ups [11, 12]. Most of them do not seek help from mental health services on their own, but are sent by adult caretakers [13], and are therefore less likely to expect benefit from treatment or to establish a trustful relationship with the therapist. Additionally, children and adolescents are known to weigh affective aspects of the therapeutic alliance higher than their caregivers do [7, 14]. Therefore, alliance ratings from children and adolescents and their caregivers or other adults may reflect different sides of a relationship and may not be interchangeable. Secondly—in contrast to adults—psychotherapy in children and adolescents requires active caregivers who, e.g. ensure regular attendance at sessions by accompanying their children to therapy, and who are willing to change their parenting behavior—if necessary—in order to enhance therapeutic success. This triangulates therapeutic relationships and creates further possibilities of therapeutic change. The active participation of caregivers is even more important in Trauma-Focused Cognitive Behavioral Therapy (TF-CBT), as caregivers are involved in each treatment session and are asked to support their children in practicing trauma-related coping skills at home. In fact, a successful involvement of caregivers has repeatedly been shown to be essential for therapeutic improvement in children and adolescents [15, 16]. Thus, results from adult studies are not well applicable to children, and the simultaneous investigation of both patients’ and caregivers’ common treatment factors is indispensable to understand their contribution to therapeutic improvement.

Although TE is considered a crucial factor for therapeutic success also with children and adolescents [17], almost no empirical research in this domain has been undertaken. In 49 children and adolescents with obsessive compulsive disorders (OCD), patients’ self-reported pre-treatment TE, but not caregivers’ TE predicted treatment response [18]. Higher TE was associated with high completion rates of exposure based Cognitive Behavioral Therapy (CBT) and symptom reduction. A similar pattern emerged in a large, multisite study about treatment for depression in adolescents. Patients’, but not parents’, TE predicted self-reported reduction of depressive symptoms immediately after treatment completion [19].

**Fig. 1:**
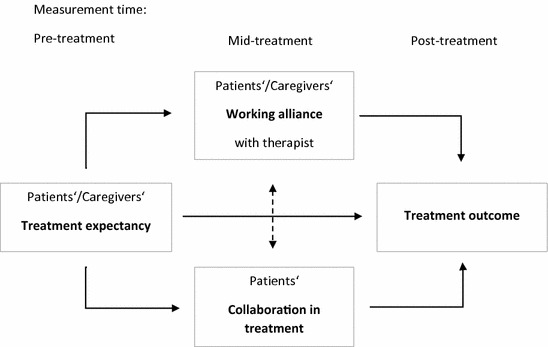
Model of treatment expectancy and other common factors in psychotherapy processes

Theoretical models trying to explain TE and its effects on therapeutic improvement often refer to the influence of other common treatment factors, such as patients’ collaboration or therapeutic alliance [20, 21]. High prognostic expectations could lead to better collaboration in therapy, e.g. regular homework compliance, and a better working alliance, thus indirectly enhancing therapeutic success (see Fig. 1). Additional common factors should be considered in a process model of therapeutic change, if one wants to understand the TE-outcome link, as these factors are shown to be associated or even to mediate the effect of expectations on therapeutic success.

Working alliance—defined as a consensus between patient and therapist regarding goals, methods and focus of the treatment [22]—might be important to understand the TE-outcome link. In adults, working alliance explains 29% of the variance of treatment outcome, regardless of the number of sessions, the type of treatment, the specificity of outcomes, or the design of the study [23, 24]. In children and adolescents, slightly smaller effects of alliance are reported (*r* = 0.14, [25]; *r* = 0.22, [26]), and some studies fail to demonstrate the alliance-outcome link [27]. With regard to children and adolescents suffering from PTSS, two randomized controlled trials (RCTs) found positive effects of therapeutic alliance on symptom reduction, especially on internalizing symptoms in the TF-CBT condition [8, 28], whereas another RCT for prolonged exposure in adolescent girls did not find any link between alliance and outcome [29]. Possibly, stronger alliance enhances collaboration and engagement in TF-CBT tasks, which leads to higher symptom reduction, but this was not investigated in children and adolescents with PTSS so far. Thus, knowledge about the association of different common treatment factors with TE and their contribution to treatment success is limited, especially regarding children and adolescents and their caregivers.

It is not clear to date, whether a positive relationship between TE and outcome in children and adolescents with depression or OCD, as well as the insignificance of this link in caregivers, can be generalized to other mental health problems, e.g. PTSS. TE may play an important role in enhancing treatment success in children and adolescents with PTSD. Moreover, caregivers are intensively involved in TF-CBT for children and adolescents, which increases the likelihood of an association of caregivers’ TE and treatment outcome. Most recent investigations of common factors in children and adolescents with PTSD focused on working alliance, neglecting TE or a more integrative model of several common factors. Most of all, recent TF-CBT studies [8, 28, 29] did not include caregivers’ rating of common factors, therefore might underestimate their important role in symptom reduction. The current study aims to fill this gap in research on TE in children and adolescents with PTSS and their caregivers. We focused on TE in TF-CBT and investigated direct effects of patients’ and caregivers’ TE on treatment outcome as well as indirect effects via working alliance and patients’ collaboration (see Fig. 1).

We examined the following hypotheses:

1. The patients’ as well as the caregivers’ TE *directly* affects patients’ treatment response to TF-CBT in terms of PTSS score, respectively PTSS reduction after treatment completion.
2. The patients’ as well as the caregivers’ TE *indirectly* affects treatment response in so far as
  - the patients’ as well as the caregivers’ TE affect patients’ collaboration *and* at the same time patients’ collaboration significantly affects patients’ treatment response;
  - the patients’ as well as the caregivers’ TE affect patients’ and caregivers’ working alliance *and* patients’ and caregivers’ working alliance affects patients’ treatment response.

In a complementary analysis, treatment outcome was operationalized by a difference score of pre- and post-treatment symptoms.

## Methods

### Patients

The present investigation was based on data collected within a randomized controlled effectiveness study (see [30] for more details of procedures and patients). Patients were consecutively recruited at eight German mental health clinics for children and adolescents according to the following inclusion criteria: a history of one or more traumatic event(s) after the age of 3 years and dating back at least 3 months; current age 7–17 years; PTSS as main mental health problem with a total symptom severity score ≥ 35 points on the Clinician Administered PTSD Scale for Children and Adolescents (CAPS-CA; [31]); sufficient knowledge of the German language to respond to questionnaires, clinical interviews and treatment; safe current living circumstances; and the co-operation of at least one non-offending caregiver. Patients with acute suicidal behavior, concurrent psychotherapy, or any change in psychotropic medication within 6 weeks before or during TF-CBT were excluded from the study. Patients whose caregivers had severe psychiatric disorders were also excluded.

Analyses of this study were undertaken with TF-CBT completers (*n* = 65), since data were only available for this subgroup (see Table 1 and [30] for more details). TF-CBT completers were predominantly accompanied by female caregivers (*n* = 49; 75%), mostly a parent or other relative (*n* = 46; 71%) instead of, e.g. an employee of the youth welfare institution. Completers of TF-CBT did not differ from participants dropping out of treatment regarding demographic or clinical variables (see Table 1). Treatment completion was defined as participation in at least 8 sessions TF-CBT (*M* = 11.9; *SD* = 1.04) and the post-treatment assessment. Within the first 8 sessions, the most stimulating components of TF-CBT—psychoeducation, relaxation and gradual exposure in sensu are scheduled to be completed [32]. Patients in the control group who received TF-CBT after completion of the waiting time were not considered for analysis.

**Table 1:** Description of the study sample

Variables	TF-CBT completers (*n* = 65)	Tf-CBT dropouts (*n* = 11)	Statistics	*p*
Female, *n* (%)	44 (67.7)	9 (81.8)	*χ*^*2*^(1) = 0.89	0.49
Age (years) *M* (*SD*; range)	12.52 (2.90; 7–17)	13.45 (3.01; 8–17)	*t*(74) = − 0.98	0.33
Living out of home, *n* (%)	15 (23.1)	0 (0)	*χ*^*2*^(1) = 3.18	0.10
Germany as birth country, *n* (%)	58 (89.2)	10 (90.9)	*χ*^*2*^(1) = 0.85	1.00
Index trauma, *n* (%)			*χ*^*2*^(1) = 1.76	0.42
Sexual violence	25 (38.5)	6 (54.5)		
Physical violence	25 (38.5)	2 (18.2)		
Other (death of a loved one, war, neglect)	15 (23.0)	3 (27.3)		
Full PTSD DSM-IV diagnosis, *n* (%)	50 (76.9)	7 (63.6)	*χ*^*2*^(1) = 0.89	0.45
≥ 1 comorbid disorder DSM-IV, *n* (%)	19 (29.2)	5 (45.5)	*χ*^*2*^(1) = 1.15	0.31
CAPS-CA total score *M* (*SD*; range) pre-treatment	57.86 (16.61; 37–102)	62.36 (22.09; 36–109)	*t*(74) = − 0.79	0.43

TF-CBT, Trauma-Focused Cognitive Behavioural Therapy; PTSD, post traumatic stress disorder; CAPS-CA, Clinician Administered PTSD Scale for Children and Adolescents

#### Treatment condition

TF-CBT is a component-based manualized treatment including parenting skills, psychoeducation, relaxation, affect modulation, cognitive processing, gradual exposure in sensu (trauma narrative) and in vivo (trauma reminders), conjoint child-caregiver sessions, and the elaboration of strategies for enhancing safety and future development (see [33] for details). Before participating in the study, therapists were carefully trained by experienced clinicians, and certified by an expert TF-CBT trainer, based on videotapes of a training case. Treatment fidelity was supported during the trial by supervision.

### Procedure

The local institutional review board approved the study, which was registered under Clinical Trials (NCT01516827). Informed consent of the parents or legal guardians, and informed assent of children and adolescents were obtained. Patients were reimbursed for their time and travel expenses to clinical assessments, but not for participating in treatment sessions. Health insurance companies covered all treatment costs.

Patients were consecutively recruited between February 2012 and January 2015 at eight German mental health clinics for children and adolescents, five of them community clinics and three located at an academic mental health care center. All clinics screened their patients; the study was additionally announced on the project’s website and on the clinics’ flyers to promote referrals.

After an initial screening for eligibility, patients and their caregivers underwent a multi-methodical baseline assessment, which comprised measurements of PTSS, other clinical and demographic variables, as well as TE of therapeutic success. TE was assessed separately in patients and their caregivers, e.g. biological parents or employees of the youth welfare system where the patient lived. Children and adolescents were randomized to either 12 sessions TF-CBT à 90 min within 16 weeks or to a waitlist of the same duration. Randomization was performed independently of the project group in a 1:1 ratio; clinics and PTSS severity were treated as strata. At mid-treatment (after 6 sessions), patients and caregivers rated their working alliance with the therapist separately, and the therapist evaluated patients’ collaboration in treatment. After treatment, patients’ PTSS and working alliances of patients and their caregivers were measured again. All assessments were made by trained, blinded, and independent evaluators. We analyzed the alliance at mid-treatment, since at an early stage of psychotherapeutic processes it proved to be a better predictor of treatment outcome than at treatment completion [23, 34].

### Instruments

The Clinician Administered PTSD Scale for Children and Adolescents (CAPS-CA) version for DSM-IV [31] was used to assess treatment outcome. Children and adolescents evaluate both the frequency and intensity of their PTSS over the last month on five-point rating scales (0 = ‘None of the time; no symptoms’ to 4 = ‘daily or almost every day; a whole lot’). Developmentally appropriate language and visual aids for the degrees of symptom frequency and intensity are used. The CAPS-CA provides a total symptom severity score with combined frequency and intensity scores (range 0–152; α = 0.79; [31]). Both the post-treatment symptom severity score and a difference score (pre-minus post-treatment symptom severity) were analyzed, the latter with higher scores indicating higher symptom reduction.

TE of patients and their caregivers was each rated by themselves by a single item with a 5-point rating scale (1 = ‘I expect this treatment to help me/my child a lot’; 5 = ‘I don’t expect this treatment to make any difference in my/my child’s condition’). The single item format is consistent with prior studies in children and adolescents [18, 19]. The scores were inversed with the result that high scores indicate high TE.

Treatment collaboration was rated by therapists by a single item on a 5-point rating scale (1 = ‘Excellent, the patient did his/her homework assiduously and actively participated during session’; 5 = ‘None, patient never finished his/her therapeutic homework and refused any participation during sessions’). To facilitate the judgment of therapists, suitable behavior examples for both ends of the scale were offered. Again, scores were inversed for analyses, and high scores therefore indicate high collaboration.

Patients and caregivers independently completed the short version of the Working Alliance Inventory (WAI-S, [35]) to rate their own alliance with the therapist, comprising 12 items with a 7-point rating scale (1 = ‘never’; 7 = ‘always’; range 12–84). The WAI is one of the most frequently used instruments with adults [36] and has also been used in research of psychotherapy with children and adolescents [29, 37]. We adapted the patient (WAI-S-P, [35]) version for children and adolescents by translating and back-translating using a systematic process based on recommendations for good practice [38]. The caregiver-therapist version (WAI-S-CT) was adapted with the same items reworded for the use by caregivers. Cronbach’s alpha for the adapted German versions total scores were 0.88 (WAI-S-P), and 0.86 (WAI-S-CT).

### Statistical analyses

Statistical analyses were performed using IBM SPSS Statistics Version 21 and Mplus Version 7.31 [39]. Variables were inspected for missing values, and single missing raw items of the WAI-S-P and WAI-S-CT were replaced by means of the other items on the respective scale of the respondent (< 1%).

To describe the study sample and to assure comparability, group differences between completers and drop-outs were tested by *t*-tests for independent samples and χ^2^ tests. In preparation of path analysis, the Kolmogorov–Smirnov test was used to test for normal distribution of variables; correlation coefficients between variables were estimated with Kendall’s *τ*, due to their skewed distribution. All statistical tests were two-tailed, and significance levels were set at *p* < 0.05.

In order to test our hypothesis, a path analysis based on structural equation modeling (SEM) was used to determine the direct and indirect effects of treatment expectancy on treatment outcome. The model was estimated with the Maximum Likelihood Robust (MLR) estimator, since the data were not normally distributed. TE served as the independent variable (IV), and working alliance, collaboration, and PTSS after treatment completion, respectively PTSS difference score as dependent variables (DV). The assumed directions of relationships in the hypothesized model are depicted in Fig. 1, correlations are indicated by lines with arrows on both ends. Path analyses were conducted and presented in accordance to guidelines [40, 41]. Model fit is perfect by definition as the model includes all possible paths between variables. Standardized parameter estimates were used for comparisons within the model.

## Results

### Preliminary analyses

Descriptive values and correlation coefficients between patients’ and caregivers’ common factors and CAPS-CA total symptom severity after completion of treatment are displayed in Table 2. None of the common variables was significantly correlated with treatment outcome (*τ* = 0.01–0.15). PTSS post-treatment, as well as common factors of patients and caregivers, were not normally distributed. The PTSS pre-post difference score was *M* = 32.31 (*SD* = 21.44).

**Table 2:** Medians, first quartiles and correlation coefficients (n = 65)

Variables	Kendall’s *τ*					Median	First quartile
	1	2	3	4	5		
1. Treatment expectancy patients	–					4.00	4.00
2. Treatment expectancy caregivers	0.18	–				4.00	4.00
3. Working alliance patients	0.33*	0.04	–			74.00	65.00
4. Working alliance caregivers	0.08	0.15	0.31*	–		78.00	72.50
5. Collaboration	0.18	0.04	0.16	0.22	–	4.00	3.00
6. Post-treatment PTSS	0.03	0.04	− 0.01	− 0.15	− 0.01	17.00	7.75

* *p* < 0.05

### Direct effects of TE on outcome

Neither the patients’ (β = − 0.026, ns; see Table 3) nor the caregivers’ TE directly predicted the treatment outcome (β = 0.183, ns). The same applies to the prediction of PTSS difference scores by patients’ (*B* = 1.042, SE *B* = 2.851, β = 0.045, *p* = 0.713) or caregivers’ TE (*B* = − 2.082, SE *B* = 5.688, β = − 0.064, *p* = 0.655).

**Table 3:** Unstandardized and standardized effects, and standard errors from path analysis

Effect	*B*	SE *B*	β	*p*
Post-treatment PTSS on				
TE patients	− 0.659	3.409	− 0.026	0.846
TE caregivers	6.418	5.429	0.183	0.221
WAI patients	0.620	0.379	0.286	0.153
WAI caregivers	− 1.946	0.493	− 0.553	0.000
Collaboration	0.999	2.945	0.039	0.732
WAI patients on				
TE patients	5.936	1.875	0.514	0.000
TE caregivers	− 0.883	2.325	− 0.055	0.694
WAI caregivers on				
TE patients	1.201	0.914	0.169	0.175
TE caregivers	1.996	1.385	0.200	0.131
Collaboration on				
TE patients	0.212	0.208	0.217	0.281
TE caregivers	0.014	0.170	0.010	0.934
WAI caregiver with WAI patients	25.564	7.091	0.446	0.000
Collaboration with WAI patients	1.732	1.352	0.217	0.234
Collaboration with WAI caregiver	1.502	0.916	0.273	0.078
TE patients with TE caregivers	0.079	0.066	0.131	0.243

TE, treatment expectancy; WAI, Working Alliance Inventory; *B*, unstandardized path coefficient; SE, standard error; β, standardized path coefficient

### Indirect effects

Neither patients’ nor caregivers’ TE had an indirect effect on PTSS score post-treatment via collaboration. TE did neither affect patients’ collaboration (β = 0.010–0.217; ns) nor did the latter predict the post-treatment outcome (β = 0.039; ns; difference score *B* = 1.061, SE *B* = 2.757, β = − 0.045, *p* = 0.697.

**Fig. 2:**
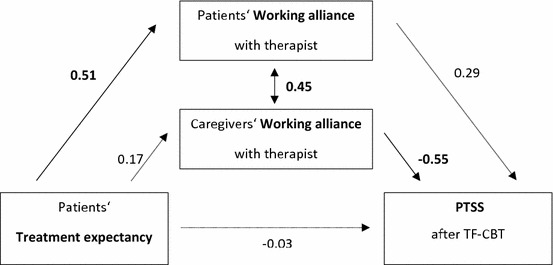
Standardized path coefficients of the model including TE, working alliance and outcome. Numbers in bold are statistically significant. PTSS posttraumatic stress symptoms; TF-CBT, Trauma-Focused Cognitive Behavioral Therapy

Patients’ TE predicted patients’ working alliance (β = 0.514, *p* < 0.001), but only caregivers’ working alliance was related to post-treatment outcome (β = − 0.533, *p* < 0.001; difference score *B* = 1.100, SE *B* = 0.522, β = 0.335, *p* = 0.031). Working alliances of patients and their caregivers were significantly correlated (β = 0.446, *p* < 0.001; see Fig. 2).

## Discussion

This study investigated direct and indirect effects of treatment expectancy on outcome of TF-CBT in children and adolescents with PTSS and their caregivers. Neither the patients’ nor the caregivers’ treatment expectancy did affect the treatment outcome directly, nor did TE affect the outcome indirectly via treatment collaboration or working alliance. These findings are confirmed when treatment outcome is defined as symptom reduction. However, caregivers’ working alliance emerged as a factor with a significant positive effect on treatment outcome.

Contrary to most findings in adults [10] and preliminary results concerning children and adolescents with OCD [18] or depression [19], treatment outcome in this TF-CBT study was not predicted by TE of patients with PTSS or their caregivers. Possibly, the TE-outcome link is less pronounced in children and adolescents compared to adult patients, which refers to a developmental effect that is also reported for the association between working alliance and treatment success [25, 42]. In comparison to adult patients, developmentally defined characteristics may limit children’s social, emotional and cognitive abilities to perceive, evaluate and report expectations and working alliance, which, as a consequence, weakens the association with symptom reduction. Alternatively, children and adolescents might have an even more vague and imprecise concept of psychotherapy than adult patients, leading to unspecific expectations which are not associated with outcome. Additionally, the intensity of the TE-outcome link might depend on whatever psychiatric disorder the patients have. It is quite conceivable that the impact of expectations might differ for patients suffering from, e.g. OCD, in comparison with children and adolescents predominantly suffering from a primary depression or PTSS. Cognitive distortions and negative expectations about oneself, the world and the future are inherent to depressive disorders and PTSS, and positive expectations regarding future treatment success may have a big impact on both. In PTSS, dysfunctional cognitions are known to be an important driver in both symptom development [43] and symptom reduction [44, 45]. Although depression is the most common comorbid condition in PTSS, knowledge of the association of these two is limited. Results point to divergent ways of therapeutic change as a function of different subtypes of comorbid PTSS and depression [46, 47]. Thus, also TE may influence treatment outcome depending on the subtype of comorbid PTSS and depression. Additionally, the conceptualization of TE as a dynamic, changeable variable seems more suitable, especially in the treatment of PTSS. Trauma-focused interventions, reported to have the best evidence for PTSS in children and adolescents [48], include the steady commitment of patients during treatment to counteract avoidance behavior. Repeated motivational techniques or psychoeducational elements may thus change TE during treatment. It is possible that TE measured later in treatment may have a stronger association with outcome than pre-treatment TE, as assessed in our study. Though, even if TE is likely to be highly influenced by the first meeting with the therapist and the presentation of the treatment model, naïve TE—i.e. TE assessed before patients ever met their therapists—was reported to be significantly associated with outcome in children and adolescents with depression or OCD in children and adolescents [19, 25] and adults [10]. Furthermore, the TE-outcome link might be more complex than we expected in our model, as associations may depend on how patients’ expectancies and therapists’ attitudes match during the first sessions [10, 49]. Also, associations might be nonlinear, with the best treatment outcome in patients with medium treatment expectations [20].

Our results are partly consistent with the well-known pathway from TE over working alliance to treatment outcome in adults [50]. Children and adolescents’ TE significantly increased their working alliance, which was positively associated with their caregivers’ working alliance and by this pathway suggests an indirect prediction of treatment outcome. Recently, the adolescents’ perception of their caregivers’ approval of TF-CBT was reported to be more important than their own alliance with the therapist to continue treatment protocol [51]. These findings emphasize the importance of caregiver participation in TF-CBT [25, 52]. Caregivers ensure a continuous treatment participation, which is especially important in PTSS, where avoidant behavior may interrupt the therapeutic exposure with traumatic memories. Therefore, caregivers willingness to actively support their child’s treatment participation is necessary to ensure treatment success [53]. Additionally, a good alliance with the therapist motivates caregivers to improve their parenting behavior, as taught in TF-CBT. This treatment component seems especially important in PTSS, as the difficulties mentioned above often challenge caregivers’ skills, leading to vicious circles of negative communication and behavior [54].

### Limitations

Several limitations apply due to the characteristics of this study. First of all, the sample size was slightly too small for investigations of TE, and statistical power was not sufficient to detect small effects of TE on outcome. However, the sample size can be regarded as sufficient for path analyses [41]. Secondly, TE was measured only once pre-treatment by a single item to avoid additional strain on patients and their caregivers, given the elaborated psychometric assessments within the study. Although former investigations [18, 19] using single items measured before start of treatment reported positive associations of TE and outcome, a more differentiated, repeated assessment of TE might have influenced results. Additionally, findings might depend on instruments, as we used an age appropriate adaptation of the WAI-S, whereas others applied, e.g. the Therapeutic Alliance Scale for Children (TASC; [55]). However, the alliance-outcome link is reported to be free from effects of the instruments used with adult patients [36], as well as with children and adolescents [25]. Moreover, ceiling effects in our variables—probably due to a positive selection of motivated study participants—limited our statistical analyses and might explain the nonsignificant findings.

## Conclusions

The influence of TE on the success of CBT in children and adolescents seems rather limited. Future studies should conceptualize TE as a dynamic construct, which may be adjusted during treatment and influence outcome together with other common factors like working alliance. TE and working alliance should be assessed repeatedly at the beginning and during psychotherapy from different perspectives, in a larger sample, and—if possible—also including patients with lower TE. Additionally, more efforts should be made to understand the role of caregivers in the treatment of PTSS in children and adolescents, as the inclusion of a supportive caregiver can be regarded as essential for therapy success in this population.

## Abbreviations

CAPS-CA: Clinician Administered PTSD Scale for Children and Adolescents
CBT: Cognitive Behavioral Therapy
OCD: obsessive-compulsive disorder
PTSS/D: posttraumatic stress symptoms/disorder
RCT: randomized controlled trial
TASC: Therapeutic Alliance Scale for Children
TE: treatment expectancy
TF-CBT: Trauma-Focused Cognitive Behavioral Therapy
WAI: Working Alliance Inventory
